# A Conceptual Framework for Studying and Investing in Precision Nutrition

**DOI:** 10.3389/fgene.2019.00200

**Published:** 2019-03-18

**Authors:** Steven H. Zeisel

**Affiliations:** Nutrition Research Institute, The University of North Carolina at Chapel Hill, Kannapolis, NC, United States

**Keywords:** precision nutrition, metabolic heterogeneity, genetic polymorphism, epigenetics, microbiome

## Abstract

Nutrients and food-derived bioactive molecules must transit complex metabolic pathways, and these pathways vary between people. Metabolic heterogeneity is caused by genetic variation, epigenetic variation, differences in microbiome composition and function, lifestyle differences and by variation in environmental exposures. This review discusses a number of these sources of metabolic heterogeneity and presents some of the research investments that will be needed to make applications of precision nutrition practical.

## Introduction

Nutrients and food-derived bioactive molecules must transit complex metabolic pathways before their ultimate use as sources of energy, catalysts for reactions, structural components, ligands for receptors or as modulators of organ function. Some of these metabolic pathways may not be human, but rather are microbial pathways. It makes sense that, between individuals, variations in the function of these complex metabolic pathways result in metabolic heterogeneity that causes differences between people in how they respond to a nutrient or bioactive molecule. Some metabolic inefficiencies will be in critical metabolic pathways, and people with these inefficiencies will develop health problems. If scientists understand the sources of this nutrition-relevant metabolic heterogeneity, they can, through computational and mathematical modeling of the underlying complex biological systems, predict these variations in responses and design interventions accordingly. In people in whom metabolic inefficiencies are predicted, clinicians could recommend diets, supplements, medical foods or pharmaceutical interventions designed to bypass or overcome these differences so as to optimize organ function and enhance health. This is the goal of the new discipline called Precision Nutrition. Precision nutrition will require that the underlying science and modeling be translated and presented so that clinicians understand the many inputs that contribute to metabolic heterogeneity-associated health problems and then recognize the appropriate precision nutrition interventions and deliver them to people with such health problems. Policy makers also will need to understand the underlying science and modeling tools, so that they can better implement precision nutrition principles in the development of policy recommendations and public health interventions.

To develop this understanding, there is a pressing need for developing enhanced methods for assessing many of these factors that contribute to metabolic heterogeneity, for better computational and informatic tools that can be used to model the complex interactions between the sources of metabolic heterogeneity in people, and for better tools to translate the science and models into tests and interventions that health professionals can implement. The ability to accomplish these precision nutrition goals will take significant investments of intellect, money and time (Table 1).

**Table 1:** Investments that are needed to advance precision nutrition.

**Diet Assessment**
1. Development of better biomarkers for assessment of diet intake 2. Development of better instruments for measuring diet intake
**Genetic Variation**
1. Development of a larger catalog of functional genetic variants 2. More studies that collect genetic, diet intake and health outcome data 3. Development of metabolomic and informatic tools for validating genetic variants that perturb metabolism in people 4. Better informatic approaches for integrating data across patterns of genetic variants
**Epigenetic Variation**
1. Development of methods for assessing epigenetic modifications in tissues that are not readily accessible in people 2. Enhanced understanding of the critical events and timing that lead to epigenetic changes that alter human metabolism. 3. Development of a larger catalog of functional epigenetic changes (that alter human metabolism)
**Microbiome Variation**
1. Development of better methods for identification of the function of microbiota in terms of effects on human metabolism 2. Inclusion of better diet intake assessment in design of microbiota studies 3. Development of better understanding of the effects of diet and exposome on microbiota populations and function
**Exposure Variation**
1. Development of better methods for identification of the exposome in people (the environmental molecules that humans are exposed to) 2. Identification of functional components of the exposome (that alter human metabolism)
**Lifestyle Variation**
1. Development of better biomarkers for assessment of lifestyle (especially physical activity) 2. Development of better instruments for measuring lifestyle (especially physical activity) 3. Development of a larger catalog of functional lifestyle variants (especially physical activity) in terms of effects on human metabolism.
**Systems Biology**
1. Development of better understanding of the interactions between all the above sources of metabolic variation 2. Development of better informatic tools for integrating data from all the above in terms of variants that perturb human metabolism
**Translation to Practice**
1. Development of better training programs for health professionals and public in precision nutrition 2. Development of precision nutrition-guided interventions for people with metabolic perturbations that cause health problems. 3. Development of informatics that can use the data defined above to identify appropriate nutrition and dietary interventions in people with metabolic perturbations that cause health problems

Obviously, we do not yet know enough about the many of the sources of metabolic heterogeneity in any individual, and much less is known about how to integrate these effects across the many sources. As always, perfection is the enemy of implementation of any approach to better health. The field is ready to implement version 1.0 of precision nutrition based on an understanding of genetic variation and nutrition and of microbiome-related variation. These first versions of precision nutrition will be imperfect but better versions will develop as the understanding of the contributions of the harder-to-measure sources of metabolic heterogeneity increases and as computational and mathematical modeling of the underlying complex biological systems advances, Gene-based prediction of metabolic heterogeneity is already in use in pharmacogenetics, an emerging area of medicine that refines drug dosing so as to match the rate that an individual metabolizes the drug (Ji et al., 2016; Pratt et al., 2018; Shukla et al., 2018; Swen et al., 2018). The enzymes involved in the metabolism of drugs (a form of bioactive molecule) did not evolve because people were exposed to these drugs, but rather evolved because people were exposed to (and needed to metabolize) components of their diets and molecules formed by their intestinal flora or by their organs. Thus, pharmacogenetics is a good guide to the first steps in developing the larger field of precision nutrition. Implementing what is already known about genetic variation and nutrition-relevant metabolic heterogeneity is the obvious next step in this process (see later discussion), but there are many steps that must follow before the full promise of precision nutrition can be achieved.

## Sources of This Nutrition-Relevant Metabolic Heterogeneity

**FIGURE 1:**
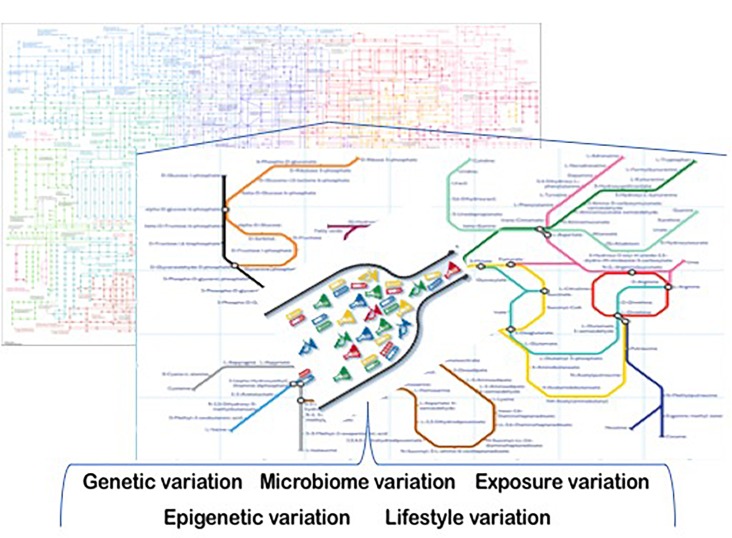
Sources of metabolic heterogeneity. Precision Nutrition is based on understanding the underlying sources of metabolic variation. These include genetic, epigenetic, microbiome, life-style and exposure-based differences among people. Some of these variations create inefficiencies (bottlenecks) in metabolism that are associated with adverse health outcomes in people who are challenged by their diets. Computational and mathematical modeling of the underlying complex biological systems that are involved, and the use of these models to develop individualized interventions are the bases for the practice of precision nutrition.

There are a broad set of factors that contribute to metabolic heterogeneity in people. Genetic variation, epigenetic variation, microbiome-related variation, lifestyle variations (e.g., physical activity, feeding behavior differences), and variation in chemical and other exposures present in a person’s environment are just a few of these factors (Figure 1). Some of these factors are better understood than others, and this review focuses on those.

## Genetic Variation as a Source of Nutrition-Relevant Metabolic Heterogeneity

Metabolism involves the function of many metabolic pathways, each step in these pathways is mediated by proteins that are coded for by genes. Codons within exons of genes determine the amino acid sequence of the protein products. In addition, the genetic code in promoter regions (usually nearby to these exons) contains response element sequences that can bind to external signals that determine whether a gene is switched on or off by regulatory enhancers such as hormones, antioxidants, calcium, metals, etc. (Resseguie et al., 2011; Hou et al., 2012). Finally, some regions of DNA do not encode for protein products, but rather for non-coding RNAs that regulate whether genes are transcribed or whether the resulting mRNA is translated into a protein (Mirra et al., 2018).

People have millions of variations in the “spelling” of their genetic code (any individual person has about 50,000 of these single nucleotide polymorphisms (SNPs) (Overbeek et al., 2005; Sabeti et al., 2007). These genetic variations are inherited from ancient ancestors, and therefore differ among people depending on their heritage (Overbeek et al., 2005; Sabeti et al., 2007). Some of these SNPs result in codon substitutions in critical genes of metabolism that change their expression, function, or half-life. Thus, variations in a person’s genetic code can affect how much of a critical metabolic enzyme (or transporter, or receptor) is available (by changing the rates of the protein’s synthesis or by changing amino acid residues that affect the half-life of the protein) and/or genetic variations can alter how well the protein works (by changing amino acid residues that affect how an enzyme binds to its substrate or that affect how the protein catalyzes the reactions involving that substrate). Such functional SNPs are one source of metabolic heterogeneity and can cause differences between people in how they respond to a nutrient or bioactive molecule.

An example of a significant metabolic difference caused by a single functional SNP is seen in women during pregnancy. Choline is an important nutrient that is needed to form a normal fetus and infant; inadequate supplies of choline lead to abnormal brain development and to birth defects (Zeisel and Blusztajn, 1994; Shaw et al., 2004, 2006; Wang et al., 2016). In addition, during pregnancy higher choline diets improve placental function (Kwan et al., 2017) and protect the mother from preeclampsia (Jiang et al., 2013). Adult men and women deprived of choline develop liver or muscle damage that resolves when choline is added to their diets (Fischer et al., 2007). In most men and post-menopausal women, choline must be ingested in the diet or they get sick, but more than half of premenopausal women can eat very low choline diets and not develop liver or muscle dysfunction (Fischer et al., 2007). Why are some premenopausal women resistant to choline deficiency? In some of these women, significant amounts of choline (in the form of phosphatidylcholine) can be formed endogenously by a reaction catalyzed by the enzyme phosphatidylethanolamine-*N*-methyltransferase (PEMT), thereby reducing these women’s dietary requirement for choline (Fischer et al., 2007). The gene *PEMT* has several estrogen response elements in its promoter region and the gene is induced by estrogen (Resseguie et al., 2007). Men and post-menopausal women don’t have enough estrogen to induce the gene’s expression, while some premenopausal women do. A very common SNP in *PEMT* (rs12325817 G > C substitution) decreases *PEMT* responsivity to estrogen thereby increasing the dietary choline requirement in premenopausal women with this SNP (Fischer et al., 2010; Resseguie et al., 2011). More than 70% of women of European heritage and of African Maasai heritage (where traditional dietary intake of choline is high) have at least one variant allele (C) of *PEMT*, while this variant is less common in women of West African descent (where traditional dietary intake of choline is low) (da Costa et al., 2006; Fischer et al., 2007, 2010; Resseguie et al., 2007; Silver et al., 2015). Premenopausal women with this *PEMT* SNP cannot make endogenous phosphatidylcholine but can bypass their metabolic inefficiency by eating more choline in their diet, so adverse effects on fetal outcome will only be detected when the SNP is combined with eating diets low in choline. It is interesting that, in the United States, intake of choline in women of pregnancy potential is significantly lower than recommended (Wallace and Fulgoni, 2016), probably because the foods that contain choline (e.g., eggs, fatty meats) often are being avoided due to dietary restriction of cholesterol intake (Zeisel et al., 2003).

There are many other examples of single gene polymorphisms that have large effects and therefore are readily apparent in studies of relatively few people. The predisposition to gain weight on a high saturated fat diet was most apparent (approximately 10% higher BMI) in people with the CC genotype of the APOA2 rs5082 (–265T > C) polymorphism and was not seen in the TT genotype (Corella et al., 2009, 2011). Another example of a significant metabolic difference caused by individual functional SNPs is seen for SNPS that influence the rates of metabolism of caffeine. Cytochrome P450 1A2 (CYP1A2) is responsible for approximately 95% of caffeine metabolism (Rasmussen et al., 2002). A functional SNP in *CYP1A2* (rs762551 A > C substitution) decreases enzyme inducibility, resulting in slower caffeine metabolism (Sachse et al., 1999) and explains the wide interindividual variability in caffeine concentrations after a caffeine dose. Similarly, individual functional SNPs in in the aryl-hydrocarbon receptor (AHR) for caffeine (rs6968865 and rs4410790) influence the wide interindividual variability in brain’s response to caffeine ingestion (the caffeine “buzz”) (Josse et al., 2012). To date, there are hundreds of functional SNPs that have been identified that exert a large enough effects so that they can be seen as metabolic heterogeneity (de Toro-Martin et al., 2017). Most of precision nutrition to date has focused on such single gene effects on nutrient metabolism.

The future of precision nutrition lies in understanding more complex patterns of genetic variation that modify metabolism. It is likely that multiple “hits” (due to functional genetic variants) in a pathway will add up to a greater perturbation of the metabolic pathway; hits in metabolically related pathways could further add to this perturbation. For example, functional SNPs in genes of folate metabolism increase the demand for methyl-donors derived from choline metabolism (Kohlmeier et al., 2005). In addition, many phenotypes are polygenic, apparent only when pathways involving multiple genes are perturbed. SNPs are not the only genetic variants that need to be considered; for example, gene copy number variation can cause functionally important perturbations in metabolism (Reiter et al., 2016; Sharma et al., 2016). For these reasons, studies advancing precision nutrition need to focus on developing computational and mathematical modeling of the underlying complex genetics and then develop algorithms for calculating the integrated effects of multiple genetic contributions that together predict metabolic inefficiencies. These algorithm-based scores can be the basis for recommending diets designed to bypass or overcome these differences so as to optimize organ function and enhance health. An example of an algorithm-based approach to precision nutrition is seen in studies of dietary choline and risk for developing fatty liver. Most men and post-menopausal women, when deprived of dietary choline develop fatty liver (da Costa et al., 2006; Fischer et al., 2007, 2010; Resseguie et al., 2007; Silver et al., 2015). This is because choline is needed to produce phosphatidylcholine needed for very-low-density-lipoprotein (VLDL) secretion from liver (Yao and Vance, 1988; Yao and Vance, 1989). People who are inefficient at exporting VLDL from liver, are prone to accumulate triacylglycerol within hepatocytes (fatty liver). There are multiple metabolic steps, each dependent on a different gene, that influence to the amount of phosphatidylcholine available to make the phospholipid envelope of VLDL. These include: transport of choline into the liver, phosphorylation of choline and the other steps involved in the Kennedy pathway for phosphatidylcholine synthesis (Kennedy and Weiss, 1956), the PEMT pathway for phosphatidylcholine synthesis (Vance et al., 1997), hydrolysis of phosphatidylcholine (Corbin et al., 2013), transport of phosphatidylcholine into bile (Davit-Spraul et al., 2010), and oxidation of choline to make metabolites that can donate methyl-groups (Corbin et al., 2013). SNPs in each of these pathways are relatively common, and an algorithm-based score that integrates the combination of effects of genetic variant-derived metabolic inefficiencies across multiple pathways accurately predicts which people will develop fatty liver as their body mass index (BMI) increases (Corbin et al., 2013).

Why haven’t genome-wide association studies identified more gene variants that are associated with metabolic inefficiencies? As discussed earlier, people can prevent the effects of metabolic inefficiencies by eating diets that are either high in content of the nutrient (for example, women who have inefficiencies in producing choline can make up for this by eating diets high in choline content), or by eating diets that provide the downstream molecules whose production is limited by the metabolic bottleneck created by the inefficiency [for example, people who have metabolic inefficiencies in producing betaine from choline can bypass this problem by ingesting betaine (Johnson et al., 2010, 2012)]. Sometimes the metabolic inefficiencies associated with a SNP are overcome by eating less. In the fatty liver pathways discussed above, SNPs cause diminished capacity for flux through the hepatic fat export pathway but this decrease does not become a problem until this capacity becomes rate limiting. When diets are low in calories and low in substrates needed for hepatic lipogenesis (lean people), genetic variants that slow the export of fat from the liver do not become rate limiting; slower export of fat from liver can still meet the slower rates of fat production (Corbin et al., 2013). It is only when people eat diets high in calories that hepatic lipogenesis exceeds the rate at which fat can be exported by liver, resulting in fat accumulation within liver; in such people the effects of the genetic variants are unmasked (Corbin et al., 2013). Thus, a functional SNP may only be revealed in people eating diets that challenge the SNP-induced metabolic inefficiency.

Finally, the ability to identify gene variations has outpaced the ability to functionally characterize them. The integration of genomic and metabolomic methods could rectify this gap by confirming that gene variants are associated with perturbations of specific metabolites in the pathway that the gene is relevant to. As discussed earlier, dietary intake must be considered in such metabolomic analyses, as gene variation may only have a functional effect under specific diet conditions. There are relatively few genetic studies that also contain good information on dietary intake and this limits the development of diet x gene interactions that can inform precision nutrition. Fortunately, the NHANES studies by the US Centers for Disease Control now collect phenotypic, genetic and dietary data (Wallace and Fulgoni, 2016). Such data sets are very valuable. It is a problem that the assessment of dietary intake is much more variable than is assessment of gene sequence (Shim et al., 2014). Computer-assisted diet intake assessment is developing nicely (Schap et al., 2014), but still, there is a pressing need for enhanced methods for assessing dietary intake. It is possible that, through measuring biomarkers, diet intake can be imputed and that this will provide a solution for improving assessment of diet intake. Studies using metabolomics to measure metabolite patterns associated with dietary patterns have promise (Menni et al., 2013; Kinross et al., 2014; Beger et al., 2016; Pallister et al., 2016; Guasch-Ferre et al., 2018). However, metabolic heterogeneity is likely to affect levels of these biomarkers, sometimes independently of dietary intake, introducing variability in the relationships between these markers and diet intake. Whatever enhanced methods become available, dietary intake data needs to be included in the big data analyses of factors that influence metabolic heterogeneity. There is a pressing need for investment in this research area.

Because a person’s genetic code stays relatively constant over the lifespan, the power of such gene-variant-based predictions is that they are measurable at any time in life, unlike most other risk factors. Excellent molecular tools are available for assessing gene sequence and genetic polymorphisms. For this reason, nutrigenetics/nutrigenomics has advanced more rapidly in making contributions to precision nutrition. However, genetic variation is only one contributor to metabolic heterogeneity.

## Epigenetic Differences as a Source of Metabolic Heterogeneity

The genetic code alone is not enough to predict phenotype. Cells in the body have highly diverse functions in different tissues and organs despite containing almost identical genetic code. These differences are due to epigenetic regulation of the genome (Ideraabdullah and Zeisel, 2018). Epigenetic regulation involves the placement of chemical marks on DNA or histones by enzymes known as writers, these marks must be recognized by specific regulator enzymes called readers that then suppress or activate gene transcription or suppress mRNA translation into protein, and ultimately the effects of these marks must be ended by enzymes called erasers that remove the epigenetic marks. To date there are two types of epigenetic marks in people: covalent modifications to DNA and covalent modifications to histone proteins.

DNA methylation is the most widely studied epigenetic mark. DNA becomes methylated by the addition of a methyl-group to cytosine to generate 5-methylcytosine (5mC). In people, 5mC occurs primarily at palindromic CG dinucleotides (Szyf, 2005). The presence of 5mC at a locus is associated with either gene repression or activation depending on location within the gene (Jones, 2012). For example, while 5mC at promoter regions is often associated with gene repression, 5mC in the gene body (introns and exons) is often associated with gene activation (Ideraabdullah and Zeisel, 2018). 5mC often does not initiate gene silencing but rather acts as a mechanism of maintaining the silenced state of a gene (Jones, 2012). DNA methylation induces metabolic heterogeneity, but conversely diet and metabolic heterogeneity influences DNA methylation (Jones, 2012). The methyl-groups used to mark DNA are derived from *S*-adenosylmethionine, which is derived from dietary methionine, 5-methyltetrahydrofolate, and betaine (from choline) and DNA methylation is directly modulated by dietary intake (Wolff et al., 1998; Szyf, 2005; Dolinoy et al., 2006; Waterland et al., 2006, 2007; Mehedint et al., 2010; Jones, 2012; Dominguez-Salas et al., 2014; Ideraabdullah and Zeisel, 2018). Methods for assessing DNA methylation have recently been reviewed (Shen and Waterland, 2007).

The nucleosome consists of eight histone proteins, two copies each of four core histones: H2A, H2B, H3, and H4, around which 147 bp of DNA is wrapped (Lai and Pugh, 2017). Histones modulate active (open) versus inactive (closed) chromatin configuration. This function is regulated by post-translational histone modifications made to the amino-terminal “tails” of histones (Strahl and Allis, 2000). These marks include methylation, acetylation, biotinylation, propionylation, succinylation, citrullination, butyrylation, glutathionylation, malonylation, formylation, crotonylation as well as ADP-ribosylation, GlcNAcylation, phosphorylation, hydroxylation and oxidation (Zhao and Garcia, 2015). Not only do histone modifications modulate genes of nutrient metabolism, many of the above marks on histones are directly derived from metabolism of nutrients and are sensitive to dietary intake (Ideraabdullah and Zeisel, 2018). For example, many histone demethylases are α-ketoglutarate-dependent dioxygenases that derive this substrate from nutrient metabolism; activity of these enzymes is also iron dependent (Kaelin and McKnight, 2013). This may explain why manipulating diet fat intake alters histone methylation (Inoue et al., 2015; Leung et al., 2016). Similar to DNA methylation, histone methylation is dependent on the availability of methyl-groups produced from dietary methyl-group donors (Strahl and Allis, 2000; Shen and Waterland, 2007; Davison et al., 2009; Mehedint et al., 2010; Jiang et al., 2012; Kaelin and McKnight, 2013; Dominguez-Salas et al., 2014; Inoue et al., 2015; Zhao and Garcia, 2015; Leung et al., 2016; Lai and Pugh, 2017). Histone acetylation is also nutrient sensitive, multiple components of the diet (such as butyrate) inhibit histone deacetylases (Rajendran et al., 2011). Some of these components are generated by gut microbiota-dependent mechanisms.

Though epigenetic marks are the most studied forms of epigenetic variation, there are also non-coding RNAs that contribute an important mechanism of epigenetic regulation of the genome (Deveson et al., 2017) and can interact with DNA, RNA, or protein to regulate gene expression, transcription and post-transcriptional activity, respectively (Holoch and Moazed, 2015). The best characterized, in terms of epigenetic function is microRNA (miR). These short non-coding RNA bind complementary mRNA targets and mark them for cleavage, degradation, or translational repression depending on the degree of base pair matching (Saetrom et al., 2007). MiR expression is sensitive to diet intake. For example, DNA methylation of miR *1451-5p* inhibits its expression (Du et al., 2016). Dietary restriction of maternal choline intake results in the overexpression of miRs in fetal brain (Trujillo-Gonzalez et al., 2018). One of these overexpressed miRs is miR129-5p; this miR prevents the formation of the epidermal growth factor receptor (EGFR) (Wang et al., 2016). Because an EGF signal is required for normal neural progenitor cell proliferation and differentiation, the low choline -induced overexpression of miR129-5p results in abnormal brain development (Trujillo-Gonzalez et al., 2018). Interestingly, many miRs are regulated at the transcriptional level by DNA methylation (Sengupta et al., 2016). As discussed earlier, DNA methylation is sensitive to dietary intake (Ideraabdullah and Zeisel, 2018). Diets high in methyl-group donors (methionine, B12, folate and choline) can increase DNA methylation of specific genes that result in a permanent change in phenotype (e.g., coat color in the *Agouti* mouse) (Waterland, 2006). DNA methylation, histone modifications and non-coding RNA are known to work together through separate but collaborative functions (Strahl and Allis, 2000) to modulate metabolic function.

Unlike the DNA sequence, epigenetic marks and non-coding RNAs are changeable during the lifespan, especially during early life, and these changes mediate the retuning of metabolism to adjust to the realities of nutrient availability and environment (Ideraabdullah and Zeisel, 2018). Thus, the timing of data collection is important. This adds a layer of complexity that will have to be dealt with as the science of precision nutrition is refined. Not only is nutrient metabolism modified by epigenetic differences, but these differences themselves are influenced by dietary intake and environment (Ideraabdullah and Zeisel, 2018).

New technology makes assessing DNA methylation and assessing epigenetic marks on histones reasonably easy (Strahl and Allis, 2000; Waterland, 2006; Saetrom et al., 2007; Shen and Waterland, 2007; Davison et al., 2009; Rajendran et al., 2011; Jiang et al., 2012; Kaelin and McKnight, 2013; Holoch and Moazed, 2015; Inoue et al., 2015; Zhao and Garcia, 2015; Du et al., 2016; Leung et al., 2016; Sengupta et al., 2016; Deveson et al., 2017; Lai and Pugh, 2017; Trujillo-Gonzalez et al., 2018). The challenge for use of epigenetics to refine precision nutrition is that there are still methodologic issues that must be overcome before scientists can effectively assess epigenetic contributions to metabolic heterogeneity. First, epigenetic marks and non-coding RNAs are usually tissue specific. As discussed earlier, these epigenetic differences direct genes to perform tissue-specific functions. Therefore, epigenetic analyses of lymphocytes usually will not provide accurate information about epigenetic marks in the target tissues that are important for metabolism (e.g., liver, muscle, brain, etc.). It is necessary to obtain a sample from the target tissue in order to obtain epigenetic analyses that reflect epigenetic regulation of that specific tissue’s gene expression, for the most part not a practical endeavor in studies of nutrition in people. At this time, there is no obvious solution to this problem. Perhaps, using imaging techniques such as *in vivo* fluorescent labeling (Beckman et al., 2018) will permit the development of non-invasive methods for the study epigenetic marks in tissues; it may be possible to use circulating cell-free DNA that is released from tissues to assess epigenetic marks within those tissues (Oussalah et al., 2018), but a great deal of research must be completed before we understand enough to use such an approach.

## Microbiome as a Source of Metabolic Heterogeneity

The gastrointestinal tract is the main route whereby people access nutrients; it is colonized by trillions of microbes (Qin et al., 2010). These microbes evolved over time to thrive in the human gastrointestinal tract, together, their genomes are ten times larger than the human genome, and they can metabolize almost any substrate that is presented to them (Goodman and Gordon, 2010). Often, these microbes access these nutrients before they are available to the people they colonize and the microbes metabolize foods and make small molecules that people then absorb (Manor et al., 2014; Hall and Versalovic, 2018). This can modulate how much energy people derive from foods (Scheithauer et al., 2016), provide people with essential vitamins [like vitamin K (Ramakrishna, 2013)], create regulator molecules such as short chain fatty acids (Ramakrishna, 2013), and modify the availability of other essential nutrients [such as choline (Zeisel and Warrier, 2017)]. People vary greatly in the microbes that they harbor (Eckburg et al., 2005), thus, the microbes populating our intestines are an important source of metabolic heterogeneity between people.

The composition of the gut microbiome is shaped predominantly by environmental factors such as diet and host genetics have a lesser role in determining microbiome composition (heritability of gut microbiome taxa is <2%). Changes in the microbiome can occur rapidly after changes in diet (David et al., 2014). The inherent complexity of the microbiome ecosystem makes assessing its contributions to metabolic heterogeneity challenging (Manor et al., 2014). The tools that are currently available for studying the microbiome are still not as well developed as the tools available for studying genetic variation. These tools have been best at telling us the names of the bacteria that are present in the microbiome [there are more than 1000 operational taxonomic units (Yatsunenko et al., 2012)]; note that current methodology still has difficulty identifying fungi in the microbiome, but better methods are being refined that can functionally characterize the microbiota in terms of metabolism. Adding up the contributions of individual species of bacteria does not accurately predict metabolism in the microbiome because of the complex web of interactions between the microbes and the human gut (Manor et al., 2014). Significant progress is being made on developing *in silico* network-based models of metabolism by the microbiome (Levy and Borenstein, 2013) but they are not yet refined enough to use to predict effects on human nutritional requirements and responses. Despite this, currently available microbiome data significantly improve the prediction accuracy for some outcomes relevant to precision nutrition compared to models that use only host genetic and environmental data (Wu et al., 2011; Yatsunenko et al., 2012; Levy and Borenstein, 2013; David et al., 2014; Korem et al., 2017; Rothschild et al., 2018). An interesting study used an algorithm that integrated blood metabolite measures, dietary habits, anthropometrics, physical activity and gut microbiota data to accurately predict variation in post-prandial glycemic response to meals between individuals (Zeevi et al., 2015).

There is a growing catalog of microbiome-related differences that modify host metabolic function. The end products of polysaccharide fermentation by gut bacteria are short-chain fatty acids (including propionate, butyrate, and acetate) and these provide an energy source for colonocytes. This contribution can represent up to 10% of daily caloric requirements (den Besten et al., 2013). Variation in microbiome composition is associated with several metabolic diseases such as obesity (Ley et al., 2005). Destruction by gut bacteria of essential nutrients can increase dietary requirements for these nutrients. For example, the presence of choline-consuming gut bacteria is associated with lower serum concentrations of choline in the host (Romano et al., 2017). Such studies suggest that, in terms of human metabolic heterogeneity, variation in the microbiome exerts a large effect, and microbiome data need to be included in the computational and mathematical modeling of the underlying complex biological systems that are developed to advance precision nutrition.

There are inherent challenges in the design of many microbiome studies. Often, the diet of the person being studied is not controlled when the microbiome is characterized (or else fasting is the only dietary condition studied). It is important that scientists develop better methods because organisms with the same name often do not express or activate the same metabolic pathways as this is dependent on the substrates they are growing in. Meta-omic methods are maturing, sequencing of the RNA in the microbes of the gastrointestinal tract will tell us which genes they have expressed (Hor et al., 2018), proteomics may tell us which proteins they are making (Jin et al., 2017), and metabolomic methods can tell us what metabolites they are producing (Lamichhane et al., 2018). The integration of these types of data with data on metabolic heterogeneity of people will eventually generate useful catalogs of microbiome-related causes of human metabolic heterogeneity. This is an important area for investment in research.

## Environmental Exposure Variation

Some of the genes and proteins important for both human and microbial metabolism are sensitive to metabolites of environmental chemicals that people are exposed to. Hormones and growth factors control metabolic pathways related to energy balance, nutrient absorption, glucose metabolism (insulin, glucagon), and lipid metabolism. The discovery of obesogenic environmental chemicals (Baillie-Hamilton, 2002) that include endocrine disrupter chemicals makes it apparent that human metabolic heterogeneity can arise because of differential exposure to environmental chemicals that modify hormonal signaling (Heindel et al., 2017). Estrogen is involved in the regulation of metabolism because this hormone modulates of food intake, body weight, glucose/insulin balance, body fat distribution, lipogenesis and lipolysis, and energy consumption. Estrogen targets POMC neurons and represses the synthesis of NPY and AgRP and thereby inhibits food intake (Asarian and Geary, 2006; Clegg, 2012; Heindel et al., 2017). Leptin interacts with estrogen to regulate energy metabolism (Roepke, 2009; Clegg, 2012). Bisphenol A (BPA), a well-studied endocrine disrupter chemical, increases weight gain and body fat after developmental exposure (Yang et al., 2016; Stojanoska et al., 2017; Amin et al., 2019) and induces insulin resistance (Heindel et al., 2017). Approximately 1000 endocrine disrupter chemicals have been described (Heindel et al., 2017), and exposure to them can result in adverse health outcomes (Heindel et al., 2015). Phthalate metabolites (used in the manufacture of plastics) activate peroxisome proliferator-activated receptors (PPAR) and thereby modify PPAR-modulated pathways in carbohydrate and lipid metabolism (Kim and Park, 2014). Finally, it is not only exposure to chemicals that needs to be considered, but exposure to environmental light as well (Versteeg et al., 2016). Most people in developed countries live in areas where the night sky is illuminated above the threshold for light pollution. Human metabolic pathways increase and decrease in activity with circadian rhythms that are modulated by light exposure. Activity/feeding and resting/fasting periods and energy expenditure are regulated by light-sensitive molecular mechanisms in the central clock that is located in the suprachiasmatic nuclei of the hypothalamus (Versteeg et al., 2016). In healthy humans, blood glucose concentrations and glucose tolerance possesses a diurnal variation, with lower glucose tolerance in the afternoon compared with the morning; insulin sensitivity of peripheral tissues and insulin secretion are both further reduced in the evening (Jarrett et al., 1972). Exposure to light pollution therefore, modifies metabolism of nutrients and changes the timing when biomarkers of metabolism need to be assessed.

Investments in understanding the interactions between environmental exposures and nutrient metabolism are needed in order to generate the data to be included in the computational and mathematical modeling of the underlying complex biological systems that describe and inform precision nutrition.

## Method Limitations and Challenges

The methods for measuring the effects of genetic variation on nutritional phenotype (nutrition-related changes in phenotype, especially health outcomes related to diet intake) are relatively mature. The major limitations derive from two sources: (1) methods for assessing diet intake are prone to errors much greater than are methods for measuring genotype or nutritional phenotype, and (2) not enough data sets exist where diet intake, genetics and nutritional phenotype are measured at the same time.

The methods for measuring the effects of epigenetic variation on nutritional phenotype are not yet refined enough for most applications in precision nutrition. There are excellent methods for measuring epigenetic variation in accessible tissues, but often these tissues are not the targets of interest relative to the changes observed in nutritional phenotype. There are very few data sets exist where diet intake, epigenetics and nutritional phenotype are measured at the same time.

The methods for measuring the effects of microbiome variation on nutritional phenotype are also limited because of difficulties in characterizing the complete microbiome (including fungi) and the metabolic functions of each of the component microbiota. These methods are developing rapidly. There are very few data sets exist where diet intake, functional characterization of microbiota and nutritional phenotype are measured at the same time.

The methods for measuring the effects of environmental variation on nutritional phenotype are available for common environmental exposures, but are limited because so few data sets exist that measure where diet intake, environmental exposures and nutritional phenotype at the same time.

Once it is possible to accurately measure all of the above, and once data sets are available from studies that measure all the above variables, the final barrier to developing precision nutrition is the understanding of the systems biology governing the interactions between all of these complex variables. To do this, mathematical models will need to be developed based on data sets that derive from experiments where diet intake, genetic, epigenetic, microbiome, environmental exposure and nutritional phenotype measures are made in the same people. Though rough approximation of such data sets can be achieved by combining studies that measure only some of the variables of interest, ultimately it will be important for funding organizations to invest in some studies that collect all the needed data so that these systems biology models can be validated.

## Translation to Practice

Precision Nutrition uses an understanding of metabolic heterogeneity and its sources in order to refine the prediction of requirements and responses to nutrients in individuals. It promises to explain why some people are responders or non-responders in diet intervention clinical trials. It will be used to refine dietary recommendations for large segments of the population, as haplotypes, diet intakes, and microbiome composition tend to be distributed similarly in large chunks of the population that share similar ancestry and lifestyles. Clinicians will be able to use precision nutrition science to provide individualized dietary recommendations, but before this can start tools will have to be created that can convert complex modeling of the systems biology of precision nutrition into recommendations for diet, diet supplement, behavioral and pharmaceutical interventions that can be offered by health professionals. Precision Nutrition is essentially a big data problem, as metabolic heterogeneity involves very complex interactions between thousands of genes, thousands of microbe taxonomic units and many different diets and environmental exposures.

## Author Contributions

SZ wrote the manuscript and was responsible for its contents.

## Conflict of Interest Statement

The author owns equity in SNP Therapeutics, a precision nutrition-focused company.
